# Intellectual and Behavioral Phenotypes of Smith–Magenis Syndrome: Comparisons between Individuals with a 17p11.2 Deletion and Pathogenic *RAI1* Variant

**DOI:** 10.3390/genes14081514

**Published:** 2023-07-25

**Authors:** Cathelijne C. Linders, Agnies M. van Eeghen, Janneke R. Zinkstok, Marie-José van den Boogaard, Erik Boot

**Affiliations:** 1 Advisium, ’s Heeren Loo, 3818 LA Amersfoort, The Netherlands; 2 Department of Genetics, University Medical Centre Utrecht, 3584 CX Utrecht, The Netherlands; 3 Department of Pediatrics, Emma Children’s Hospital, Amsterdam, University Medical Center, 1105 AZ Amsterdam, The Netherlands; 4 Department of Psychiatry, Radboud University Medical Centre, 6500 HB Nijmegen, The Netherlands; 5 Karakter Child and Adolescent Psychiatry, 6501 BB Nijmegen, The Netherlands; 6 Department of Psychiatry and Brain Center, University Medical Center Utrecht, 3584 CG Utrecht, The Netherlands; 7 The Dalglish Family 22q Clinic, University Health Network, Toronto, ON M5G 2C4, Canada; 8 Department of Psychiatry & Neuropsychology, Maastricht University, 6200 AB Maastricht, The Netherlands

**Keywords:** Smith–Magenis syndrome, 17p11.2 deletion, pathogenic *RAI1* variant, behavioral problems, intellectual disability, rare disorders

## Abstract

Aim: Smith–Magenis syndrome (SMS) is a rare genetic neurodevelopmental disorder caused by a 17p11.2 deletion or pathogenic variant in the *RAI1* gene. SMS is associated with developmental delay, intellectual disability (ID), and major sleep and behavioral disturbances. To explore how genetic variants may affect intellectual functioning and behavior, we compared intellectual and behavioral phenotypes between individuals with a 17p11.2 deletion and pathogenic *RAI1* variant. Method: We reviewed available clinical records from individuals (aged 0–45 years) with SMS, ascertained through a Dutch multidisciplinary SMS specialty clinic. Results: We included a total of 66 individuals (*n* = 47, 71.2% with a 17p11.2 deletion and *n* = 19, 28.8% with a pathogenic *RAI1* variant) for whom data were available on intellectual functioning, severity of ID (*n* = 53), and behavioral problems assessed with the Child Behavior Checklist (CBCL, *n* = 39). Median full-scale IQ scores were lower (56.0 vs. 73.5, *p* = 0.001) and the proportion of individuals with more severe ID was higher (*p* = 0.01) in the 17p11.2 deletion group. Median total CBCL 6–18 scores (73.5 vs. 66.0, *p* = 0.02) and scores on the sub-scales somatic complaints (68.0 vs. 57.0, *p* = 0.001), withdrawn/depressed behavior (69.5 vs. 55.0, *p* = 0.02), and internalizing behavior (66.0 vs. 55.0, *p* = 0.002) were higher in the *RAI1* group. Conclusion: The results of this study suggest that 17p11.2 deletions are associated with a lower level of intellectual functioning and less internalizing of problems compared to pathogenic *RAI1* variants. The findings of this study may contribute to personalized-management strategies in individuals with SMS.

## 1. Introduction

Smith–Magenis syndrome (SMS) is a rare genetic neurodevelopmental disorder, estimated to be present in 1:15,000–25,000 births [1,2]. SMS is caused by a 17p11.2 deletion or a pathogenic variant in the retinoic acid induced 1 gene (*RAI1*), which is located within the 17p11.2 chromosomal region [3,4], and has been shown to be responsible for most SMS features [5]. Other genes may play a role in the variability and severity of the phenotype [5]. The syndrome is associated with several physical and other manifestations, including developmental delay, intellectual disability (ID), sleep disturbances, obesity, and behavioral problems, although the expression may differ from individual to individual [6,7]. Clinical phenotyping summaries on SMS can be found in GeneReviews^®^ [8].

Previous studies have reported that the majority of individuals with SMS have ID, most often moderate or mild ID [9,10,11,12], which is characterized by significant limitations in intellectual (i.e., full-scale IQ scores < 70), adaptive, and everyday executive functioning [13,14]. In addition, many individuals with SMS are reported to exhibit problematic externalizing (e.g., aggression and self-injury) and internalizing (e.g., anxiety, depression, and somatic complaints) behavior that pose a substantial burden to patients and their families, and negatively effects their quality of life [12].

However, research examining the effects of the disease-causing genetic variants on intellectual and behavioral phenotypes in SMS is scarce, and previous studies have typically been performed on individuals with a 17p11.2 deletion. For example, a report on 48 individuals with a 17p11.2 deletion suggested that individuals with large deletions (>3.7 Mb) were more likely to have lower full-scale IQ (FSIQ) scores and lower levels of adaptive behavior functioning compared to those with small (<3.7 Mb) or common 17p11.2 deletions (3.7 Mb) [15]. Knowledge about intellectual functioning in individuals with a pathogenic *RAI1* variant has been limited to a few individuals reported in the literature [10,16,17]. Strikingly, these individuals had relatively high FSIQ scores compared to what has been reported in individuals with a 17p11.2 deletion.

Similarly, there is a paucity of literature on behavioral problems in individuals with a pathogenic *RAI1* variant, and thus little is known about to what extent the available knowledge of SMS is applicable to those with such a genetic mutation [8]. In a previous study on 31 children and adults with a 17p11.2 deletion and 10 with a pathogenic *RAI1* variant, attention seeking and self-injurious behavior were reported in the majority of patients, based on parental report [18]. No differences between the two groups were observed in these behaviors. In another study on 105 individuals with SMS that collected patient data by report (e.g., parent surveys, educational evaluations, and specialist reports), those with a pathogenic *RAI1* variant (*n* = 10) were reported to show more polyembolokoilamania (insertion of hands or objects into mouths or other body openings), skin picking, self-hugging, overeating issues, and obsessive-compulsive tendencies, compared to those with a 17p11.2 deletion [19].

In this study, we aimed to address the knowledge gap on the relationship between genetic variants and intellectual functioning and behavioral problems in SMS, by systematically comparing phenotypes between individuals with a 17p11.2 deletion and pathogenic *RAI1* variant.

## 2. Methods

### 2.1. Study Design and Setting

This retrospective cohort study was based on a comprehensive review of patient records in an outpatient sample of individuals with SMS [20]. The setting was a national multidisciplinary clinic for children, adolescents, and adults with SMS at ’s Heeren Loo, a large healthcare organization for individuals with intellectual disabilities in The Netherlands. In this clinic, ID physicians, behavioral specialists, speech therapists, dieticians, and occupational therapists provide clinical practice recommendations to parents, caregivers, and healthcare professionals on SMS-associated morbidities.

Patients were referred by their pediatrician, clinical geneticist, general practitioner, or ID physician. The study was approved by the Institutional Review Board of Amsterdam UMC in The Netherlands (#W20_098).

### 2.2. Characterization of Individuals with SMS

We ascertained individuals with molecularly confirmed SMS who were referred to our clinic between 2002 and 2020. We systematically collected relevant and anonymized clinical data on each individual, including information on demographic characteristics (age at most recent assessment, age at genetic diagnosis, and sex), the results of genetic testing reports (including FISH, microarray, and WES data) to ascertain genetic profiles (referring to a 17p11.2 deletion or pathogenic *RAI1* variant, as well as deletion size and variant type, respectively), and intellectual and behavioral phenotypes. Individuals with no data on intellectual functioning and/or behavioral problems were not included in the study.

### 2.3. Full-Scale IQ and Intellectual Disability

Available FSIQ scores were collected from official psychometric test reports in patient records. The presence or absence, and the severity, of ID, were determined based on all information on intellectual functioning and adaptive behavior (covering conceptual, social, and practical domains) in lifetime clinical records, school reports, and collateral history from family members, in addition to FSIQ scores, and according to the *Diagnostic and Statistical Manual of Mental Disorders*, fifth edition (DSM-5) [21].

We dichotomized the ID data in borderline/mild ID and moderate/severe ID to be able to perform statistical analysis, given low proportions of individuals with borderline ID (total cohort), and moderate and severe ID in individuals with a pathogenic *RAI1* variant.

### 2.4. Behavioral Questionnaires

Data regarding behavioral problems were recorded through review of available Child Behavior Checklist (CBCL) data: the CBCL 1.5–5 for children aged 1.5 to 5 years and the CBCL 6–18 for children and adolescents aged 6 to 18 years [22,23]. These questionnaires, containing 99 and 113 items, respectively, and three-point Likert-scales, were completed by parents or primary caregivers of the individual with SMS. CBCL questionnaires contain empirically based (CBCL 1.5–5) or syndrome sub-scales (CBCL 6–18), internalizing and externalizing behavior sub-scales, and total scores. For analyses, we used age- and sex-adjusted T-scores. Scores of 70 or higher on one of the empirically based/syndrome sub-scales, and 64 or higher on the internalizing, externalizing, or total problems scales, were classified as ‘clinical’, indicating psychopathology [22,23].

### 2.5. Statistical Analyses

We used Fisher’s exact tests for categorical and Mann–Whitney U tests and Spearman’s rank correlations for continuous data, given the relatively small sample sizes and asymmetric data distribution. Judgement as to whether continuous variables were normally distributed was based on an integral assessment of the information from descriptive statistics and normality plots. We used the Benjamini–Hochberg procedure to correct the analyses for multiple comparisons. In addition to between-group comparisons (17p11.2 deletion vs. *RAI1* group) in CBCL scores, we compared the proportion of patients with scores in the clinical range between both groups. We calculated Spearman’s rank correlations between FSIQ scores and scores on the CBCL 6–18 for those domains that were statistically significantly different between both groups (17p11.2 deletion vs. *RAI1* group), in order to get an impression as to what extent these differences could reflect an IQ, rather than a true genotype, effect. All analyses were two-tailed, with statistical significance defined as *p* < 0.05, and performed in IBM SPSS software (Statistics 22; SPSS, Inc., Chicago, IL, USA).

## 3. Results

Demographic data are presented in Table 1. For a total of 66 individuals (aged 2–45 years), data on intellectual (*n* = 53, 80.3%) and/or behavioral phenotypes (*n* = 39, 59.1%) were available. Forty-seven individuals (71.2%) had a 17p11.2 deletion: twenty-one (44.7%) a 3.7 (≥3.3, ≤3.8) Mb deletion, three (6.4%) a small deletion (1.1, 1.4, and 2.1 Mb, respectively), and three (6.4%) a large deletion (all 4.8 Mb). For 20 (42.6%) individuals, the deletion size was unknown. Nineteen individuals (28.8%) had a pathogenic *RAI1* variant: fifteen (78.9%) frameshift, three (15.8%) nonsense, and one (5.3%) unknown. Details on the *RAI1* mutations per study participant (i.e., nucleotide change, protein change, and type of mutation) are provided in Supplementary Table S1. There were no significant between-group differences in age at last assessment, children <18 years, or sex. Median age at genetic SMS diagnosis was significantly higher in the group of individuals with a pathogenic *RAI1* variant.

### 3.1. Full-Scale IQ and Intellectual Disability

**Figure 1:**
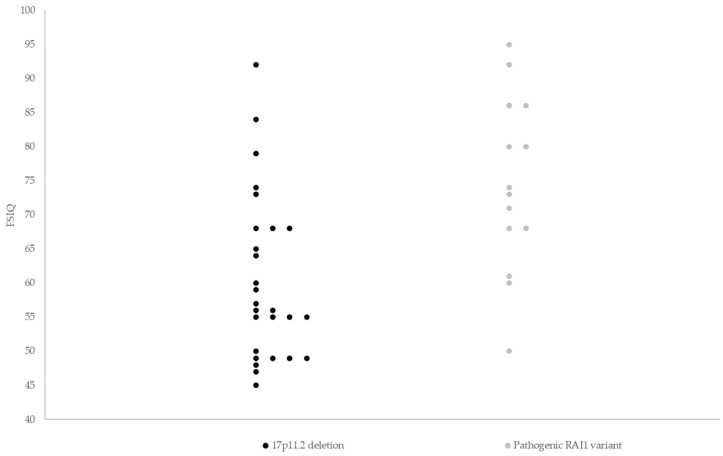
Full-scale IQ scores of 41 individuals with Smith–Magenis syndrome. Median FSIQ scores were lower in the group of individuals with a 17p11.2 deletion (56.0, range 45–92) compared to individuals with a pathogenic *RAI1* variant (73.5, range 50–95, *p* = 0.001). Horizontal lines indicate median FSIQ scores. FSIQ = full-scale intelligence quotient.

FSIQ data were available for 41 individuals (Figure 1). Although the proportion of individuals who had IQ data available was lower in those with a 17p11.2 deletion (*n* = 27, 57.4%) than in individuals with a pathogenic *RAI1* variant (*n* = 14, 73.7%), this difference was not statistically different (*p* = 0.40).

**Figure 2:**
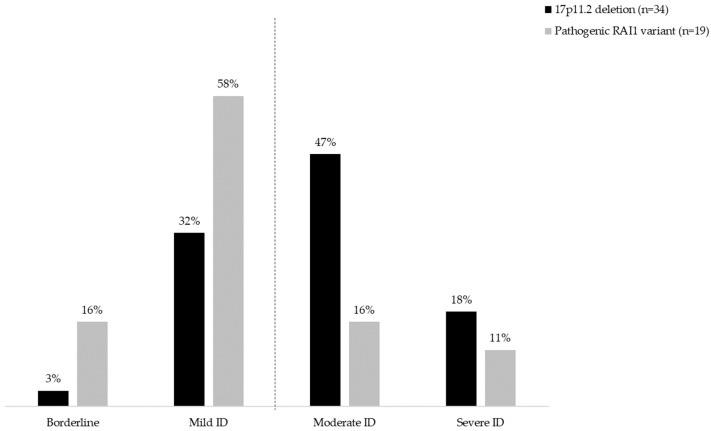
ID severity in 53 individuals with Smith–Magenis syndrome. The dotted line represents a line to divide the individuals with borderline/mild from those with moderate/severe ID. The proportion of individuals with moderate/severe ID was higher in the 17p11.2 deletion group (22 out of 34; 64.7%) than in the group with a pathogenic *RAI1* variant (5 out of 19; 26.3%, *p* = 0.01). ID = intellectual disability.

Median FSIQ scores were lower in those with a 17p11.2 deletion (56.0, range 45–92) compared to individuals with a pathogenic *RAI1* variant (73.5, range 50–95, *p* = 0.001). The proportion of moderate/severe ID was higher in individuals with 17p11.2 deletion (22 out of 34; 64.7%) than in those with a pathogenic *RAI1* variant (5 out of 19; 26.3%, *p* = 0.01, Figure 2).

### 3.2. Behavioral Questionnaires

Visual representations of CBCL scores on an individual level are provided in Table 2 (CBCL 6–18) and Supplementary Table S2 (CBCL 1.5–5). CBCL 6–18 data were available for 24 individuals, including 10 with a pathogenic *RAI1* variant (Table 3). CBCL 1.5–5 data were available for 17 individuals, 2 with a pathogenic *RAI1* variant (Supplementary Table S3). Median total CBCL 6–18 scores and scores on the sub-scales withdrawn/depressed behavior, somatic complaints, and internalizing behavior, were higher in the *RAI1* group than in the 17p11.2 deletion group. Results for somatic complaints and internalizing behavior remained significant after Benjamini–Hochberg correction for multiple testing. A higher proportion of patients in the *RAI1* group compared to the 17p11.2 deletion group had scores in the clinical range on the CBCL 6–18 sub-scales of withdrawn/depressed behavior (50% vs. 0%, *p* = 0.006), somatic complaints (40% vs. 0%, *p* = 0.02), and internalizing behavior (70% vs. 7%, *p* = 0.002). Results for withdrawn/depressed behavior and internalizing behavior remained significant after Benjamini–Hochberg correction for multiple testing.

### 3.3. Relationship between FSIQ Scores and CBCL 6–18 Scores

Eighteen individuals (*n* = 8 with a pathogenic *RAI1* variant) had FSIQ and CBCL 6–18 data. For this sub-sample, there were strong statistically significant positive correlations between FSIQ scores and the CBCL 6–18 sub-score somatic complaints (r_s_ = 0.68, *p* = 0.002), and moderate statistically significant positive correlations between FSIQ scores and internalizing behavior (r_s_ = 0.59, *p* = 0.01), which may suggest an effect of IQ on CBCL 6–18 scores. However, no such correlations were found in the sub-group analyses: somatic complaints (17p11.2 deletion, r_s_ < 0.01, *p* = 0.99 vs. pathogenic *RAI1* variant r_s_ = 0.13, *p* = 0.77) and internalizing behavior (17p11.2 deletion, r_s_ = −0,16, *p* = 0.67 vs. pathogenic *RAI1* variant r_s_ = 0.32, *p* = 0.45). Correlations between FSIQ scores and withdrawn/depressed behavior were not statistically significant (r_s_ = 0.37, *p* = 0.13).

## 4. Discussion

To the best of our knowledge, this study is the first to systematically compare FSIQ scores and behavioral questionnaires in a sample of individuals with a 17p11.2 deletion and a pathogenic *RAI1* variant. The study adds to the scarce body of literature on genotype–phenotype correlations in SMS.

In line with previous reports in smaller uncontrolled samples [10,15,16,17], median FSIQ scores were higher in the group of individuals with a pathogenic *RAI1* variant, in comparison with those with a 17p11.2 deletion. However, the range in FSIQ scores in both groups was wide, i.e., 45–92 in the 17p11.2 group and 50–95 in the *RAI1* group. This suggests that variation in FSIQ scores may be significant regardless of genetic subtype. This variability has also been reported in studies that included individuals with a 17p11.2 deletion only [12,15]. We notice that, in our study, median FSIQ scores (56, range 45–92) in individuals with a 17p11.2 deletion were somewhat higher than reported in a previous study in children and adults (48, range 41–81) [10]. Possible explanations for this finding include differences between the intelligence scales used and in who was or was not selected for taking a specific intelligence test. Another explanation may be that individuals assessed in earlier studies may have had a more severe SMS phenotype on average, due to the fact that genetic testing for SMS (i.e., FISH) was less available clinically and required an index of suspicion in the early years, potentially resulting in a selection bias towards a more severe phenotype. Previously reported FSIQ scores in a total of 10 children and adults with a pathogenic *RAI1* variant ranged from 57 to 89 [10,16,17], with a median of 67.5, in line with findings in this study.

The proportion of individuals with more severe ID was lower in the *RAI1* group than in the 17p11.2 deletion group. In the *RAI1* group, about three quarters had borderline to mild ID. In individuals with a 17p11.2 deletion, the majority had mild to moderate ID (about 50% moderate ID), similar to what has been reported earlier [10,12]. A challenge in comparison with earlier research, in addition to the paucity of reports in individuals with a *RAI1* variant [16], is that historically, ID severity was mainly based on intellectual functioning. Classification of ID has changed over time, with adaptive functioning (collection of conceptual, social, and practical skills) have become now equally important [24].

Remarkably, while externalizing behaviors are common in both genetic subtypes (based on this and previous studies) [7,12], the results of this study suggest that 17p11.2 deletions are associated with less internalizing problems, i.e., behavioral problems directed toward oneself such as withdrawal and depression, and somatic complaints, as compared to pathogenic *RAI1* variants. Only one (7%) individual with a 17p11.2 deletion had clinical internalizing behavior scores in the clinical range, compared to >41 in previous studies [7,12]. Clinical externalizing behavior in individuals with a 17p11.2 deletion (50%) is lower compared to previous studies (82%) [7,12]. Although we do not have an explanation for this discrepancy, it may suggest relative under-reporting of internalizing behaviors in individuals with a 17p11.2 deletion in our CBCL 6–18 data (in contrast to those of the CBCL 1.5–5), which may also have driven the differences between internalizing behavior problems in individuals with a 17p11.2 deletion compared to those with a pathogenic *RAI1* variant. How to understand the high proportion of individuals with a pathogenic *RAI1* variant and scores in the clinical range on the domain internalizing behaviors? Individuals with a pathogenic *RAI1* variant were diagnosed at a later age compared to individuals with a 17p11.2 deletion. In addition, to direct genetic effects, this may have impacted the natural course of and accessibility to services. SMS requires multidisciplinary management, including psycho-education, parental guidance, and specific treatments [25]. If these are not provided, a less favorable course with respect to mental health could be hypothesized. In addition, it is conceivable that individuals with a pathogenic *RAI1* variant suffer more from asynchronies between their cognitive and emotional development, with emotional maturity delayed beyond intellectual functioning, which may lead to internalizing behaviors [8]. Individuals with a pathogenic *RAI1* variant typically are emotionally ‘young’ compared to their cognitive level of functioning [5,26]. Although one might think that higher levels of intellectual functioning would go along with better verbal and mentalizing (reflective) skills, which may lead to less internalizing behavioral problems [27], we did not find a correlation of FSIQ scores with scores on the CBCL either in the 17p11.2 deletion or *RAI1* group. Future research is needed to better understand these probably much more complex relationships.

## 5. Implications and Future Directions

Our findings, collectively, have implications for psycho-education and management of individuals with SMS, and their relatives. Historic SMS literature tended to focus on externalizing behaviors, such as self-injurious and aggressive behavior, and on hyperactivity, attention problems, and stereotypic behavior [6]. In line with recent reports [7,12], this study also shows internalizing behaviors in SMS. We recommend that internalizing behavior should also be considered, especially in those with a pathogenic *RAI1* variant. In choosing the right school, adults and teachers should not only focus on the cognitive abilities of the child, which may be relatively high, in particular in those with a pathogenic *RAI1* variant, but should also pay attention to social, communicative, and practical skills and emotional development. Research into the potential role of the aforementioned asynchrony in cognitive and emotional functioning in individuals with SMS, needs consideration. We argue that future studies on SMS should preferably stratify by genetic variant, or at least provide clarity on the genetic information of the study participants, to enable interpretation of the study results. Well-powered prospective studies will be needed to optimize personalized approaches that take differences into account regarding cognitive, emotional, and adaptive functioning, and the risk of developing both externalizing and internalizing behaviors.

## 6. Strengths and Limitations

This study has the largest number of individuals with a pathogenic *RAI1* variant to date, which enabled systematic comparisons between individuals with 17p11.2 deletions and pathogenic *RAI1* variants. A limitation is the retrospective nature of the study. Although age differences were taken into account by using T-scores, and although there was no statistically significant between-group difference in age, we cannot rule out an effect of age on the study findings. In addition, the age range was large, especially for studying behavior, including both children and adults. When interpreting the FSIQ findings, account must be taken that, for some individuals with SMS, no scores were available, due to the fact that their cognitive impairments made it impossible to complete an intelligence test with a FSIQ score. We included scores for individuals with significant discrepancies between (non-) verbal and performance IQ scores (17p11.2 deletion *n* = 3, pathogenic *RAI1* variant *n* = 2) to maximize use of available data, even though FSIQ scores based on such discrepant IQ profiles may not be clinically reliable on an individual level. Another limitation is that quantitative data on FSIQ and adaptive functioning were not available or could not be determined in young children. In addition, in this study, information on adaptive functioning was not based on one standardized questionnaire. Finally, the fact that only a very few individuals were known to have a small or large 17p11.2 deletion, hampered our ability to test for any effect of deletion size.

## 7. Conclusions

This study identified differences in the intellectual and behavioral phenotypes of SMS and suggests that pathogenic *RAI1* variants are associated with relatively higher FSIQ scores and more internalizing behaviors than 17p11.2 deletions. Prospective longitudinal studies are required to validate and refine these findings to better inform the clinical implications.

## Figures and Tables

**Table 1:** Demographics of 66 individuals with Smith–Magenis syndrome. Bold font indicates statistical significance. ^a^ Mann–Whitney U tests for continuous data and Fisher’s exact tests for categorical data. IQR = interquartile range, y = years.

	Total *n* = 66		17p11.2 Deletion *n* = 47		*RAI1* Variant *n* = 19		Statistics ^a^
	*n*	%	*n*	%	*n*	%	*p*
Female	34	51.5	22	46.8	12	63.2	0.28
Children (<18 y)	52	78.8	36	73.5	16	84.2	0.74
	Median	IQR	Median	IQR	Median	IQR	*p*
Age at diagnosis, y	5.0	9.0	4.0	5.0	11.0	9.0	**0.0003**
Age at last assessment, y	12.5	9.0	10.0	10.0	14.0	5.0	0.08

**Table 2:** Heatmap depicting CBCL 6–18 scores in 24 individuals with Smith–Magenis syndrome.

Demographics	17p11.2 Deletion															*RAI1* Variant											Analyses
Subject #	41	11	46	62	3	29	40	25	72 ^a^	28	32	35 ^a^	17	57	%	33	13	22	73	60	31	78	65	80	83		
Deletion size, Mb/type of *RAI1* variant	-	3.7	3.7	-	1.4	3.7	3.6	3.8	2.8	-	-	-	3.3	-		fs	fs	fs	ns	fs	fs	fs	fs	fs	fs		
Sex	m	m	m	f	m	f	m	f	f	m	f	m	m	f		m	m	f	m	m	f	f	f	m	f		
Age, y	6	6	6	6	7	8	9	9	9	10	10	13	13	15		7	8	8	8	9	12	11	12	17	14		
**Syndrome scales (50–100)**															**% ^b^**											**% ^b^**	***p* ^c^**
Anxious/depressed	53	50	50	54	66	60	50	57	51	50	50	54	57	55	0	51	62	65	57	57	74	50	57	50	68	10	0.42
Withdrawn/depressed	54	50	66	52	68	52	66	52	52	54	56	57	66	60	0	58	76	56	73	76	63	52	66	75	78	50	**0.006**
Somatic complaints	57	57	53	66	57	53	50	57	50	50	57	58	58	56	0	68	74	68	61	74	76	57	68	61	76	40	0.02
Attention problems	69	59	71	77	83	62	67	73	68	71	64	71	67	68	43	79	75	62	69	67	87	66	77	65	83	50	1
Aggressive behavior	64	65	61	76	73	59	64	69	51	72	65	73	66	65	28	83	72	66	62	89	92	50	75	61	70	60	0.21
Social problems	60	65	62	72	60	62	62	67	59	78	59	69	67	66	14	73	69	59	69	78	80	59	69	69	61	30	0.62
Thought problems	67	67	67	75	75	58	64	73	58	73	70	75	76	70	57	84	79	62	73	73	88	58	73	78	79	80	0.39
Rule-breaking behavior	53	53	50	63	57	55	53	59	50	60	63	64	64	54	0	72	57	55	57	70	80	50	60	64	68	3	0.06
**Domain scales**															**% ^b^**											**% ^b^**	***p* ^c^**
Internalizing behavior (34–100)	54	45	52	59	67	56	52	56	46	45	48	57	62	58	7	61	72	64	66	71	73	48	66	63	74	70	**0.002**
Externalizing behavior (33–100)	61	62	56	72	70	58	61	67	47	70	65	69	67	62	50	76	69	66	61	77	83	44	69	63	69	70	0.42
Total CBCL score (24–100)	64	61	63	72	73	60	65	70	54	71	64	71	69	67	71	77	76	64	70	78	86	55	72	71	75	90	0.36

Bold font indicates statistical significance, meeting the threshold for multiple comparisons with Benjamini–Hochberg correction. T-score (total, internalizing, externalizing): <60 = normal range (white), 60–63 = borderline range (light brown), ≥64 = clinical range (brown). T-score (syndrome scales): <65 = normal range (white), 65–69 = borderline range (light brown), ≥70 = clinical range (brown). ^a^ Two individuals also had CBCL 1.5–5 data (Shown in Supplementary Table S1). ^b^ Proportion of individuals with a clinical score. ^c^ Fisher’s exact tests for comparisons of proportions between those with a 17p11.2 deletion and a pathogenic *RAI1* variant. CBCL = Child Behavior Checklist, fs = frameshift, ns = nonsense, m = male, f = female.

**Table 3:** Age- and sex-adjusted T-scores on the CBCL 6–18 in 24 individuals with Smith–Magenis syndrome.

	Total *n* = 24		17p11.2 Deletion *n* = 14		*RAI1* Variant *n* = 10		Analyses	
Syndrome scales	Median	IQR	Median	IQR	Median	IQR	Effect size ^a^	*p* ^b^
Anxious/depressed behavior	54.5	9.0	53.5	7.0	57.0	15.0	−0.33	0.10
Withdrawn/depressed behavior	59.0	15.0	55.0	14.0	69.5	19.0	−0.49	0.02
Somatic complaints	57.5	12.0	57.0	5.0	68.0	14.0	−0.75	**0.001**
Attentional problems	69.0	10.0	68.5	5.0	72.0 ^c^	14.0	−0.17	0.41
Aggressive behavior	66.0	11.0	65.0	9.0	71.0 ^c^	23.0	−0.23	0.25
Social problems	66.5	9.0	63.5	8.0	69.0	14.0	−0.27	0.18
Thought problems	73.0 ^c^	9.0	70.0 ^c^	9.0	75.5 ^c^	10.0	−0.36	0.08
Rule-breaking behavior	58.0	11.0	56.0	10.0	62.0	14.0	−0.37	0.07
Domains								
Internalizing behavior	58.5	14.0	55.0	11.0	66.0 ^c^	10.0	−0.64	**0.002**
Externalizing behavior	66.5 ^c^	9.0	63.5	9.0	69.0 ^c^	14.0	−0.27	0.19
Total CBCL scores	70.0 ^c^	9.0	66.0 ^c^	9.0	73.5 ^c^	9.0	−0.47	0.02

Bold font indicates statistical significance; i.e., meeting the threshold for multiple testing with Benjamini–Hochberg correction. ^a^ Effect sizes were determined by the z-score, divided by the root of the total number of samples. ^b^ Mann–Whitney U tests. ^c^ Scores in the clinical range. CBCL 6–18 = Child Behavior Checklist for children aged 6 to 18 years, IQR = interquartile range.

## Data Availability

The data are not publicly available due to ethical restrictions and privacy concerns. Any data requests can be directed to the corresponding author.

## Supplementary Materials

The following supporting information can be downloaded at: https://www.mdpi.com/article/10.3390/genes14081514/s1, Table S1: *RAI1* mutations in 19 patients with a pathogenic *RAI1* variant. Table S2: Heatmap depicting CBCL 1.5–5 T-scores in 17 individuals with Smith–Magenis syndrome. Table S3: CBCL 1.5–5 scores in 17 individuals with Smith–Magenis syndrome.
