# Supplementary material for: Intellectual and Behavioral Phenotypes of Smith–Magenis Syndrome: Comparisons between Individuals with a 17p11.2 Deletion and Pathogenic RAI1 Variant
Source: Genes (Basel). 2023 Jul 25;14(8):1514. doi: 10.3390/genes14081514 (PMC10453904; doi:10.3390/genes14081514)
Supplement: Supplementary file 1 [file genes-14-01514-s001.zip › genes-2505576-supplementary.pdf]

**Supplementary Table S1**: *RAI1* mutations in 19 patients with a pathogenic *RAI1* variant.

| Patient | Nucleotide change | Protein change | Type of mutation |
|---|---|---|---|
| 1 | c.456_457insTGCC | p.(Pro153Cysfs*86) | frameshift |
| 2 | c.2966_2969del | p.(Lys989Serfs*74) | frameshift |
| 3 | c.2650_2651delAG | p.(Arg884Alafs*59) | frameshift |
| 4 | c.3702delC | p.(Lys1235Argfs*80) | frameshift |
| 5 | c.3103dupC | p.(Gln1035Profs*31) | frameshift |
| 6 | c.3002delG | p.(Arg1001Leufs*63) | frameshift |
| 7 | c.518delT | p.(Val173Alafs*79) | frameshift |
| 8 | c.3103dup | p.(Gln1035Profs*31) | frameshift |
| 9 | unknown | unknown | unknown |
| 10 | c.2730dupC | p.(Lys911Glnfs*33) | frameshift |
| 11 | c.1810C>T | p.(Gln604*) | nonsense |
| 12 | c.363G>A | p.(Trp121*) | nonsense |
| 13 | c.5297_5298delAG | p.(Gln1766Argfs*21) | frameshift |
| 14 | c.3103dupC | p.(Gln1035Profs*31) | frameshift |
| 15 | c.2610delG | p.(Glu870Aspfs*80) | frameshift |
| 16 | c.3421C>T | p.(Gln1141*) | nonsense |
| 17 | c.3103dupC | p.(Gln1035Profs*31) | frameshift |
| 18 | c.2257del | p.(Asp753Metfs*66) | frameshift |
| 19 | c.3142del | p.(Ala1048Profs*16) | frameshift |

**Supplementary Table S2:** Heatmap depicting CBCL 1.5-5 T-scores in 17 individuals with Smith-Magenis syndrome.

| **Demographics** | **17p11.2 deletion** | | | | | | | | | | | | | | | | ***RAI1* variant** | | |
|---|---|---|---|---|---|---|---|---|---|---|---|---|---|---|---|---|---|---|---|
| Subject # | 24 | 14 | 45 | 72 [a] | 74 | 10 | 35 [a] | 36 | 44 | 79 | 16 | 39 | 20 | 76 | 84 | | 64 | 69 | |
| Deletion size, Mb/type of *RAI1* variant | 3.4 | 3.5 | - | 2.8 | 3.7 | 2.1 | - | 3.7 | 3.7 | 4.8 | 3.3 | - | 1.2 | 3.5 | 3.7 | | ns | ns | |
| Sex | m | m | f | f | m | f | m | m | m | m | m | m | m | m | f | | f | f | |
| Age, y | 1 | 2 | 2 | 2 | 2 | 3 | 3 | 3 | 3 | 3 | 4 | 4 | 5 | 5 | 5 | | 5 | 5 | |
| **Empirically based scales (50-100)** | | | | | | | | | | | | | | | | % [b] | | | % [b] |
| Anxious/depressed | 51 | 50 | 56 | 50 | 59 | 56 | 52 | 52 | 52 | 50 | 50 | 51 | 70 | 50 | 50 | 6 | 69 | 56 | 0 |
| Withdrawn | 56 | 56 | 63 | 63 | 79 | 67 | 60 | 56 | 67 | 51 | 61 | 60 | 63 | 56 | 56 | 6 | 76 | 56 | 50 |
| Somatic complaints | 50 | 65 | 58 | 53 | 68 | 72 | 50 | 74 | 53 | 62 | 50 | 68 | 58 | 53 | 53 | 13 | 65 | 53 | 0 |
| Attention problems | 67 | 50 | 73 | 77 | 77 | 77 | 62 | 73 | 73 | 57 | 73 | 67 | 73 | 70 | 70 | 67 | 73 | 73 | 100 |
| Aggressive behaviour | 58 | 50 | 72 | 50 | 88 | 77 | 64 | 69 | 66 | 75 | 65 | 75 | 84 | 56 | 65 | 40 | 70 | 82 | 100 |
| Sleep problems | 64 | 51 | 82 | 50 | 70 | 59 | 56 | 62 | 59 | 59 | 56 | 62 | 62 | 67 | 56 | 13 | 64 | 70 | 50 |
| Emotionally reactive | 55 | 50 | 77 | 59 | 80 | 87 | 69 | 77 | 69 | 55 | 70 | 67 | 77 | 55 | 65 | 40 | 87 | 77 | 100 |
| **Domain scales** | | | | | | | | | | | | | | | | % [b] | | | % [b] |
| Internalizing (29-100) | 51 | 49 | 67 | 55 | 74 | 79 | 60 | 70 | 64 | 51 | 55 | 73 | 71 | 49 | 55 | 47 | 76 | 65 | 100 |
| Externalizing (28-100) | 60 | 40 | 74 | 56 | 86 | 81 | 64 | 71 | 69 | 70 | 68 | 70 | 82 | 60 | 67 | 73 | 73 | 80 | 100 |
| Total CBCL score (28-100) | 59 | 43 | 75 | 56 | 85 | 74 | 64 | 70 | 68 | 64 | 64 | 64 | 78 | 59 | 63 | 67 | 82 | 73 | 100 |

Bold font indicates statistical significance, meeting the threshold for multiple comparisons with Benjamini-Hochberg correction. T-score (total, internalizing, externalizing): <60=normal range (white), 60-63=borderline range (light brown), ≥ 64=clinical range (brown). T-score (syndrome scales): <65=normal range (white), 65-69=borderline range (light brown), ≥70=clinical range (brown). [a] Two individuals also had CBCL 6-18 data (Shown in Figure 1). [b] Proportion of individuals with a clinical score. CBCL=Child Behavior Checklist, m=male, f=female, ns=nonsense.

**Supplementary Table S3:** CBCL 1.5–5 scores in 17 individuals with Smith-Magenis syndrome

| | Total group n=17 | | 17p11.2 deletion n=15 | | *RAI1* variant n=2 | |
|---|---|---|---|---|---|---|
| Empirically based scales | Median | IQR | Median | IQR | Median | IQR |
| Anxious/depressed behaviour | 52.0 | 6.0 | 51.0 | 6.0 | 62.5 | - |
| Withdrawn behaviour | 60.0 | 9.0 | 60.0 | 7.0 | 66.0 | - |
| Somatic complaints | 58.0 | 14.0 | 58.0 | 15.0 | 59.0 | - |
| Attentional problems | [a] 73.0 | 6.0 | [a] 73.0 | 6.0 | 73.0 | - |
| Aggressive behaviour | 69.0 | 15.0 | 66.0 | 17.0 | [a] 76.0 | - |
| Sleep problems | 62.0 | 10.0 | 59.0 | 8.0 | 67.0 | - |
| Emotionally reactive behaviour | 69.0 | 20.0 | 69.0 | 22.0 | [a] 82.0 | - |
| Domains | | | | | | |
| Internalizing behaviour | 64.0 | 19.0 | 60.0 | 20.0 | [a] 70.5 | - |
| Externalizing behaviour | [a] 70.0 | 15.0 | [a] 69.0 | 14.0 | [a] 76.5 | - |
| Total CBCL scores | 64.0 | 14.0 | 64.0 | 15.0 | [a] 77.5 | - |

[a] Scores in the clinical range. CBCL 1.5-5=Child Behavior Checklist for children aged 1.5 to 5 years, IQR=interquartile range.
